# Citric Acid Tunes the Formation of Antimicrobial Melanin-Like Nanostructures

**DOI:** 10.3390/biomimetics4020040

**Published:** 2019-05-30

**Authors:** Pietro Melone, Giuseppe Vitiello, Michela Di Napoli, Anna Zanfardino, Maria Federica Caso, Brigida Silvestri, Mario Varcamonti, Gerardino D’Errico, Giuseppina Luciani

**Affiliations:** 1 Department of Chemical, Materials and Production Engineering, University of Naples “Federico II”, p.le V. Tecchio 80, 80125 Naples, Italy; pietro.melone@unina.it (P.M.); brigida.silvestri@unina.it (B.S.); 2 CSGI, Center for Colloid and Surface Science, via della Lastruccia 3, 50019 Sesto Fiorentino (FI), Italy; gerardino.derrico@unina.it; 3 Department of Biology, University of Naples “Federico II” Via Cintia 4, I-80126 Naples, Italy; michela.dinapoli@unina.it (M.D.N.); anna.zanfardino@unina.it (A.Z.); mario.varcamonti@unina.it (M.V.); 4 Nanofaber Spin-Off at Italian National Agency for New Technologies, Energy and Sustainable Economic Development (ENEA), Casaccia Research Centre, Via Anguillarese 301, 00123 Rome, Italy; maria.federica.caso@gmail.com; 5 Department of Chemical Sciences, University of Naples “Federico II” Via Cintia 4, I-80126 Naples, Italy

**Keywords:** hybrid nanostructure, melanin-like, citrate–Ti complex, antimicrobial, biomimicry

## Abstract

Nature has provided a valuable source of inspiration for developing high performance multifunctional materials. Particularly, catechol-containing amino acid l-3,4-dihydroxyphenylalanine (l-DOPA) has aroused the interest to design hybrid multifunctional materials with superior adhesive ability. DOPA oxidative polymerization mediated by either melanogenic enzymes or an alkaline environment involving catechol intermolecular cross-linking, ultimately leads to melanin oligomers. Recently, relevant studies disclosed the ability of Ti-based nanostructures to tune melanin’s supramolecular structure during its formation, starting from melanogenic precursors, thus improving both antioxidant and antimicrobial properties. In this work, we propose a novel biomimetic approach to design hybrid DOPA melanin-like nanostructures through a hydrothermal synthesis opportunely modified by using citric acid to control hydrolysis and condensation reactions of titanium alkoxide precursors. UV-Vis and Electron paramagnetic resonance (EPR) spectroscopic evidences highlighted the key role of citrate–Ti(IV) and DOPA–Ti(IV) complexes in controlling DOPA polymerization, which specifically occurred during the hydrothermal step, mediating and tuning its conversion to melanin-like oligomers. Trasmission electron microscopy (TEM) images proved the efficacy of the proposed synthesis approach in tuning the formation of nanosized globular nanostructures, with high biocide performances. The obtained findings could provide strategic guidelines to set up biomimetic processes, exploiting the catechol-metal complex to obtain hybrid melanin-like nanosystems with optimized multifunctional behavior.

## 1. Introduction

Nature has provided a valuable source of inspiration for developing high-performance multifunctional materials [1,2,3]. Notably, amino acid l-3,4-dihydroxyphenylalanine (l-DOPA) has been exploited to produce a huge number of biocompatible materials for many biomedical applications. Actually, the presence of catechol-groups can confer to DOPA-based materials superior surface adhesion properties, which are highly recommended in tissue adhesives [4,5,6]. Ideally, bioadhesives are supposed to be multifunctional biocompatible materials, combining wet tissue adhesion with innate antimicrobial action. Actually, site infections can impair wound healing, leading to chronic lesions [4]. Indeed, weak adhesion strength, poor biocompatibility and lack of any antimicrobial activity in currently available bioadhesives hugely limits their applications [4].

DOPA oxidative polymerization mediated by melanogenic enzymes or an alkaline environment involves catechol intramolecular cross-linking and can basically lead to melanin oligomers, following the melanogenic pathway [5]. This class of hydrophobic pigments combines impressive adhesion strength with intrinsic multifunctional behavior that account for their numerous biological roles, ranging from photoprotection, metal ion chelation, free radical quenching, as well as innate antimicrobial and anti-inflammatory efficacy [7,8,9].

Recently, relevant studies disclosed the ability of inorganic nanostructures to tune melanin’s supramolecular structure during its formation, starting from melanogenic precursors, and thus, ultimately improving physico-chemical [10], antioxidant and antimicrobial properties [9,11,12,13]. In this route, TiO_2_ sols proved to mimic melanogenic enzymes activity, driving 5,6-dihydroxyindole-2-carboxylic acid (DHICA) oxidative polymerization to melanin through DHICA–Ti(IV) ligand to metal charge transfer complex (LMCTC) [14]. This biomimetic approach led to hybrid nanostructures with unique antimicrobial activity, even under visible light [9,11,12]. However, this strategy proved ineffective in promoting DOPA cyclization and further conversion into melanin, rather than producing DOPA-based polyphenols. As a result, obtained hybrid nanostructures showed relevant antioxidant activity, but poor antimicrobial efficacy.

Among bioavailable species with assessed antimicrobial efficacy, attention has been recently focused on citric acid [15], a naturally-derived organic compound available in citrus fruits. Produced as an intermediate in the Krebs cycle in the metabolism of all aerobic organisms, citric acid is nontoxic and biocompatible [16]. Furthermore, citrate-based materials show significant antibacterial ability, which is ascribed to the high number of free carboxyl groups that can lower the local pH, avoid the nicotinamide adenine dinucleotide (NADH) oxidation, and/or change the cell membrane permeability through metal ions chelating ability, causing its damage [17]. In this work, we propose a novel biomimetic approach to design hybrid DOPA melanin-like nanostructures through a hydrothermal synthesis opportunely modified by using citric acid to control hydrolysis and condensation reactions of titanium alkoxide precursors. Spectroscopic evidences obtained by Electron Paramagnetic Resonance (EPR) and UV-Vis analysis highlighted the key role of citrate–Ti(IV) and DOPA–Ti(IV) complexes in controlling DOPA polymerization, also mediating and tuning its conversion to melanin-like oligomers. This ability was confirmed by Transmission Electron Microscopy (TEM), proving the efficacy in controlling nanostructures size and morphology. Furthermore, short-term biocide performance was assessed on Gram(−) *Escherichia coli* strains.

Overall, these findings will provide strategic guidelines to set up biomimetic processes, exploiting the catechol–metal complex to obtain hybrid melanin-like nanosystems with optimized multifunctional behavior in myriad application fields from electronics to electrocatalysis, biosensors and biomedical systems.

## 2. Materials and Methods

### 2.1. Materials

l-3,4-Dihidroxyphenylalanine (DOPA), titanium isopropoxide (TTiP), anhydrous citric acid, isopropanol anhydrous, and triethylamine (TEA) were purchased from Sigma-Aldrich (Milan, Italy). All chemicals were used as received. 5,6-dihydroxyindole-2-carboxylic acid (DHICA) monomer was prepared as described elsewhere [18,19].

### 2.2. Synthesis of Hybrid Melanin-Like Nanostructures

Hybrid melanin-like nanostructures were prepared via a hydrothermal synthetic route, adapting a previous protocol to a more eco-friendly bioinspired approach [9,20]. DOPA precursor and titanium isopropoxide were used to synthesize the hybrid organic/inorganic nanostructures. Briefly, the inorganic precursor solution was obtained by adding dropwise 6 mL of a 1.69 M solution (Sol-1) of TTiP in anhydrous isopropanol to 31 mL of a 1 M water solution of citric acid at pH 1.5 (Sol-2), kept under vigorous stirring. The white precipitate obtained after Sol-1 addition dissolves completely within 2 days, leading to the formation of a stable aqueous solution composed by a Ti–citrate complex. Subsequently, 50 mg of DOPA was added and allowed to react for 5 min. The reaction mixture was finally neutralized by a dropwise addition of triethylamine up to pH = 7.0. The obtained dark orange suspension was sealed in a Teflon recipient (the liquid volume corresponding to 75% of the whole), placed into a circulating oven, and kept at 120 °C for 24 h. The DOPA amount was chosen accordingly to the same amount of melanin precursor used in the previous studies, which gave the best antimicrobial activity [12]. This synthetic procedure was analogously repeated by using a DHICA monomer as a reference system, considering that it is produced by DOPA cyclization in melanogenic pathway. All final hybrid nanostructures were recuperated by centrifugation (11,000 rpm for 25 min) and repeated washing (three times) with distilled water. Obtained samples were named DOPAmel-like and DHICAmel-like nanostructures, respectively.

### 2.3. Quantitative Determination of Melanin Content

Each melanin-like nanoparticle suspension (100 µL at 4 mg/mL) was dispersed in 1 mL of 1 M NaOH and 3% H_2_O_2_ and heated in a boiling water bath for 30 min. After cooling, pale yellow solutions were transferred to micro-test tubes and cleared by filtration. Supernatants were analyzed with a Shimadzu UV-2600 spectrophotometer (Shimadzu Italia, Milan, Italy) determining the absorbance values at 350 nm. Melanin contents were estimated by the average of three independent measurements.

### 2.4. Physico-Chemical Characterization

Ultraviolet-visible (UV-Vis) absorption spectra on the reactive mixtures were recorded with a Cary 100 UV-Vis spectrometer (Agilent, Rome, Italy) from 300 to 700 nm, placing the sample into 1 cm path-length quartz optical cuvettes. The estimated resolution was 1 nm and the background was corrected with Milli-Q water.

Electron Paramagnetic Resonance (EPR) measurements were carried out at ~25 °C by means of X-band (9 GHz) Bruker Elexys E-500 spectrometer (Bruker, Rheinstetten, Germany), equipped with a super high-sensitivity probe head. The analyzed samples were put into flame-sealed glass capillaries which were coaxially inserted in a standard 4 mm quartz sample tube. The spectra were acquired by using the following instrumental settings: sweep width of 120 G; modulation frequency of 100 kHz; modulation amplitude of 1.0 G; and resolution of 1024 points. The amplitude of the field modulation was preventively checked to be low enough to avoid detectable signal over-modulation. By using these acquisition parameters, EPR spectra were permitted to specifically monitor the organic component. Specifically, two sets of EPR measurements were acquired: The former was performed on the aqueous reacting mixtures to monitor the DOPA and DHICA evolution before the hydrothermal treatment, while the second one was realized on the solid powders obtained after the hydrothermal step, in order to investigate the chemical and structural properties of the organic moiety within the final nanosystems. EPR spectra of liquid samples were registered with a microwave power of 0.24 mW and several scans, typically 64, were accumulated to improve the signal-to-noise ratio. Instead, EPR spectra of solid ones were registered with a microwave power of 0.06 mW to avoid microwave saturation of the resonance absorption curve and 16 scans were accumulated to improve the signal-to-noise ratio. Power saturation curves were also recorded by varying the microwave power from 0.004 mW to 128 mW. For each sample, 12 different spectra corresponding to distinct values of incident powers were collected. The quantitative analysis (i.e., *g*-factor and spin-density values) of EPR spectra was realized by means of an internal standard composed by Mn^2+^/MgO powder, inserted in the quartz tube co-axially with the analyzed samples [21]. On the other hand, the quantitative analysis of the EPR spectra was realized by determining the signal line width, ΔB, measured as the peak-to-peak distance of the first-derivative signal (instrumental output), while the determination of the Gaussian and Lorentzian contributions to the line-shape was obtained by estimating the ∆B_1/2_/∆B ratio, where ∆B_1/2_ is the half-height width of the EPR absorption signal. In all the cases considered in the present work, the line shape features were estimated and reported as percentages of the Lorentzian character.

The weight loss of the dried gels as well as the nature and temperatures of the various reactions occurring while heating the samples were evaluated by a TA Instrument simultaneous thermoanalyser (SDT Q600, TA Instrument-Waters s.p.a., Milan, Italy), following an experimental procedure as previously described [22,23]. The Thermogravimetric/differential thermal analysis (TG/DTA) tests were performed on 20 mg dried gel specimens in air (temperature range: from room temperature to 800 °C; heating rate: 10 °C·min^−1^).

The morphology of prepared hybrid nanostructures was investigated by carrying out Transmission Electron Microscopy analysis. Particularly, samples for TEM analysis were prepared by placing a drop of the hybrid suspensions on one side of the copper grids, 200 mesh with a carbon membrane. TEM images were obtained with a TECNAI 20 G^2^: FEI COMPANY (CRYO-TEM-TOMOGRAPHY, Hillsboro, OR, USA) microscope equipped with an Eagle 2HS camera: HT 120 KV; camera exposure time: 1 sec; size 2048 × 2048 pixels.

### 2.5. Antimicrobial Assays

The antimicrobial activities of DOPAmel-like and DHICAmel-like nanostructures were evaluated against *Escherichia coli* DH5α. A single colony of this strain was resuspended in 5 mL of Luria-Bertani (LB) broth and incubated overnight at 37 °C. When the culture reached an OD600 of 1 unit, it was diluted 1:100 in phosphate buffer (20 mM, pH 7.0). Samples were prepared by adding 1/25 volume of bacterial cells and nanostructure suspensions were used at fixed concentrations of 200 µg/mL; 500 µL final volume was reached with phosphate buffer (20 mM, pH 7.0). Negative control was represented by untreated cells. Samples were incubated at 37 °C for 4 h; two dilutions (1:100 and 1:1000) of all samples were placed on solid medium LB agar and incubated overnight at 37 °C. Standard deviations were less than 5% for each experiment (which was performed at least in triplicate).

## 3. Results and Discussion

### 3.1. Spectroscopic Investigation on Hybrid Melanin-Like Nanostructures

**Figure 1:**
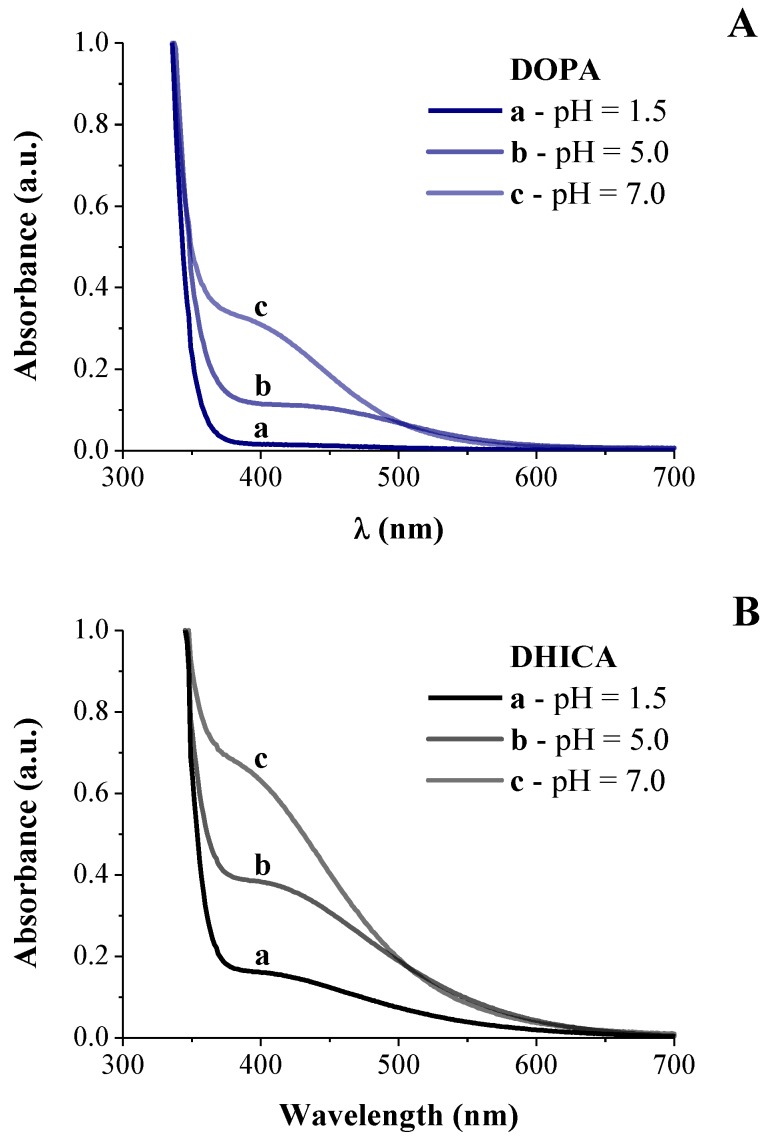
UV-Vis spectra of Ti–citric acid solution in the presence of DOPA (**A**) and DHICA (**B**) precursors.

First, a spectroscopic analysis was carried out on the aqueous reacting mixtures, alternatively containing DOPA or DHICA precursors. Particularly, a spectroscopic UV-Vis analysis was performed during the neutralization process in the first stage of the synthesis, with the aim to study the molecular mechanism of monomers’ evolution in the presence of Ti–citric acid solution. The recorded UV-Vis spectra are shown in Figure 1.

UV-Vis spectrum of Ti–citric acid solution with DOPA at pH = 1.5 (Figure 1A, spectrum a) showed an adsorption edge at about λ = 350 nm, which is ascribable to the metal–citrate complex [24], thus suggesting the formation of a Ti–citrate specie in the reactive mixture at low pH value. Raising pH to 5.0, the solution turned into orange/red, thus suggesting the formation of the Ti–catechol complex, featuring an absorption band at λ ~ 435 nm (Figure 1A, spectrum b), which shifted at λ = 400–420 nm, suggesting l-DOPA oxidation (at pH = 7, Figure 1A, spectrum c) [1,25,26]. Furthermore, no new absorption bands peaking at ca. 475 nm, associated to the characteristic electronic transition of the colorful oxidized states of l-DOPA, were observed (Figure 1A), indicating that the intramolecular cyclization did not occur. As expected, the catechol-Ti complex formation was pH-sensitive and not stable at pH values lower than 5.0 [1], thus confirming that no polymerization reactions of the DOPA precursor started.

This behavior was not appreciated in the case of the DHICA precursor (Figure 1B). In fact, after DHICA addition to the reactive mixture at pH = 1.5, the solution rapidly turned into dark red, due to the formation of the DHICA–Ti complex absorbing in the visible light (λ > 420 nm) as shown in Figure 1B. Then, starting from pH = 5, the solution turned into brown and concurrently a UV broad band absorption in the whole visible range was observed, confirming that DHICA polymerization started.

**Figure 2:**
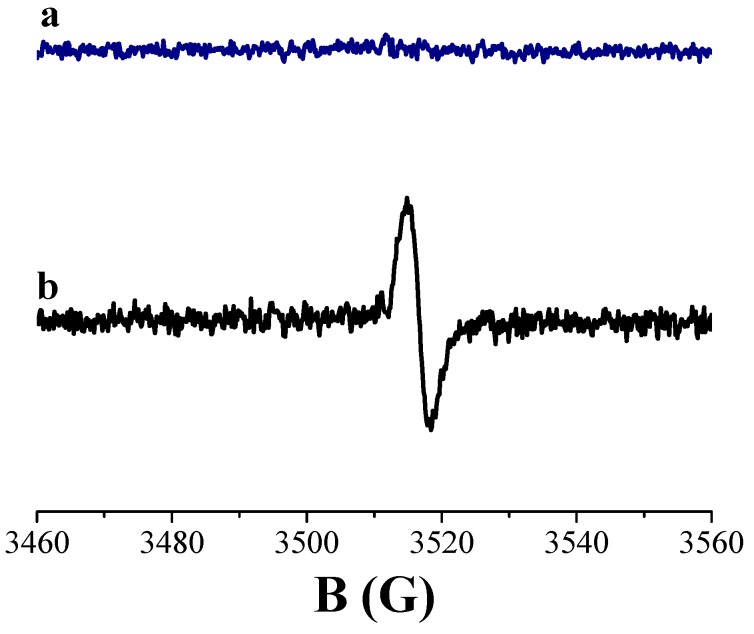
Electron paramagnetic resonance (EPR) spectra of Ti–citric acid sol reactive mixtures at pH = 7 in the presence of l-3,4-dihydroxyphenylalanine (l-DOPA) (**a**) and 5,6-dihydroxyindole-2-carboxylic acid (DHICA) (**b**) precursors.

These results were also confirmed by EPR spectra acquired during this synthesis stage. When the DOPA monomer was added to the aqueous Ti–citric acid sol, no peak appeared in the EPR spectrum at pH = 7 (Figure 2a), which is different to the roughly symmetric signal observed in the spectrum recorded for the brown Ti–citric acid sol containing the DHICA monomer at pH = 7 (Figure 2b). This central peak was centered at a *g*-factor value of ~2.0030 ± 0.0003, which is typical of carbon-centered radicals in polyindolic systems, featuring different oxidation states, forming the melanin backbone as reported in the literature [27,28,29].

More specifically, this peak was not ascribable to the DHICA monomer which is silent to EPR since it does not present unpaired electrons. However, this signal showed similar features to that previously revealed for DHICA–melanin produced by enzymatic polymerization as well as for hybrid DHICA/TiO_2_ nanostructures [9,11,12], confirming that DHICA polymerization started even before the hydrothermal treatment, differently from what happened to the Ti–DOPA system.

After 24 h of hydrothermal treatment, intense dark brown suspensions were obtained for both DOPA-based and DHICA–based systems. From a structural point of view, the absence of any diffraction peak in the X-ray diffraction (XRD) profiles of both samples proved their amorphous structure (Figure S1), differently from that observed for previously prepared eumelanin/TiO_2_ nanosystems [9]. At the same time, TG analysis also allowed to assess the amount of organic components in the final systems (Figure S2), resulting equal to about 90% *w*/*w* for both DOPAmel-like and DHICAmel-like samples. Furthermore, the Fourier-transform infrared (FTIR) spectra (Figure S3) of both samples lack bands at 485 cm^−1^ and 732 cm^−1^ featuring O–Ti–O bonding vibrations in the TiO_2_ structure [30]. Thus, XRD, FTIR and TG results supported the evidence that TiO_2_ did not form during the hydrothermal stage. This must be due to the Ti(IV)–citrate binding that hindered the hydrolysis and condensation reactions of Ti(IV) precursor, ultimately inhibiting its precipitation to titanium hydroxide and its consequent evolution into TiO_2_ nanocolloidal suspension during the hydrothermal process.

An estimation of the polyindolic content within the hybrid nanostructures was obtained by a spectrophotometric assay [31]. Nanoparticles were dispersed in alkaline media under oxidizing conditions to get the conversion of polyindolic systems into pyrrole carboxylic acids whose determination was quantitatively estimated by absorbance intensity at 350 nm. Comparing experimental results obtained from DOPAmel-like and DHICAmel-like nanostructures (Figure S4) with the calibration curve obtained starting from pure DHICA and DOPA melanins [32], an average melanin contents of 3.5 ± 0.1 mg/mL for DHICAmel-like nanostructures and 3.3 ± 0.1 mg/mL for the DOPAmel-like ones, respectively, were disclosed. This result clearly indicated that the content of melanin-like oligomers in both nanostructures was comparable, as expected since the DOPA monomer was allowed to oxidize/cyclize without any constraints at the aminoacidic side chain. However, the slightly lower amount of detected pyrrol-carboxylic acids may be ascribed to the presence of partially uncyclized DOPA units which cannot give pyrrolic acids as degradation products. These evidences were in agreement with the TG analysis, indicating the prevalent content of the organic component.

**Figure 3:**
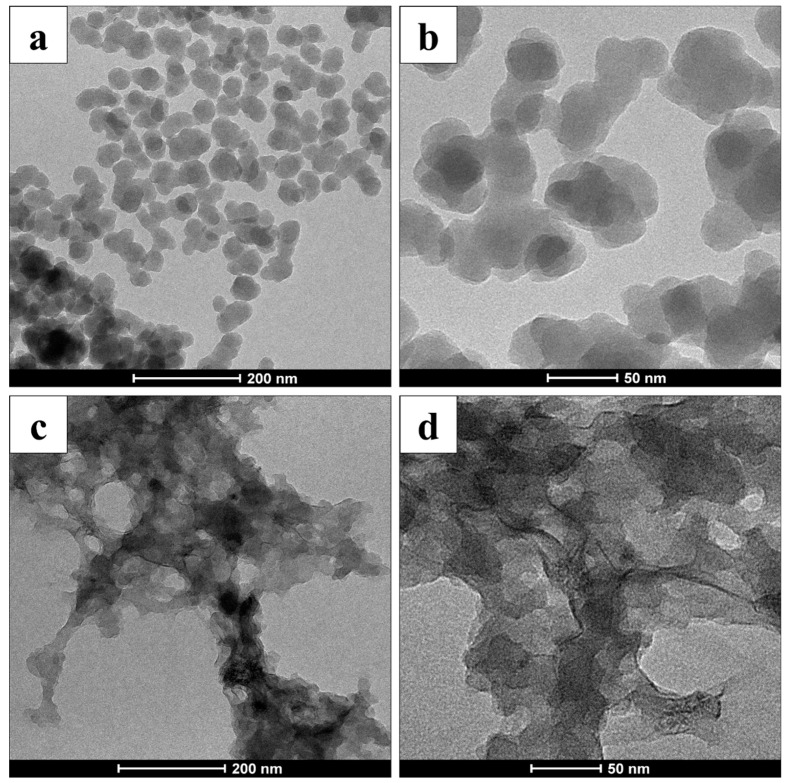
Transmission Electron Microscopy of hybrid DOPAmel-like (**a**,**b**) and DHICAmel-like (**c**,**d**) nanostructures.

Transmission Electronic Microscopy analysis shed light on the morphology of the obtained hybrid DOPAmel-like and DHICAmel-like systems. As shown in Figure 3, a globular structure with a diameter of about 30 nm was observed for the DOPAmel-like sample, while a “flakes-like” morphology was appreciated for the DHICAmel-like one. TEM images clearly supported the different polymerization pathways of the two organic precursors in the presence of the citrate–Ti complex. Slower growth kinetics involving DOPAmel-like nanostructures allowed their self-structuring into a uniform morphology and size. In contrast, a faster DHICA polymerization process caused a random organization of the pigment, resulting in an irregular shape.

**Figure 4:**
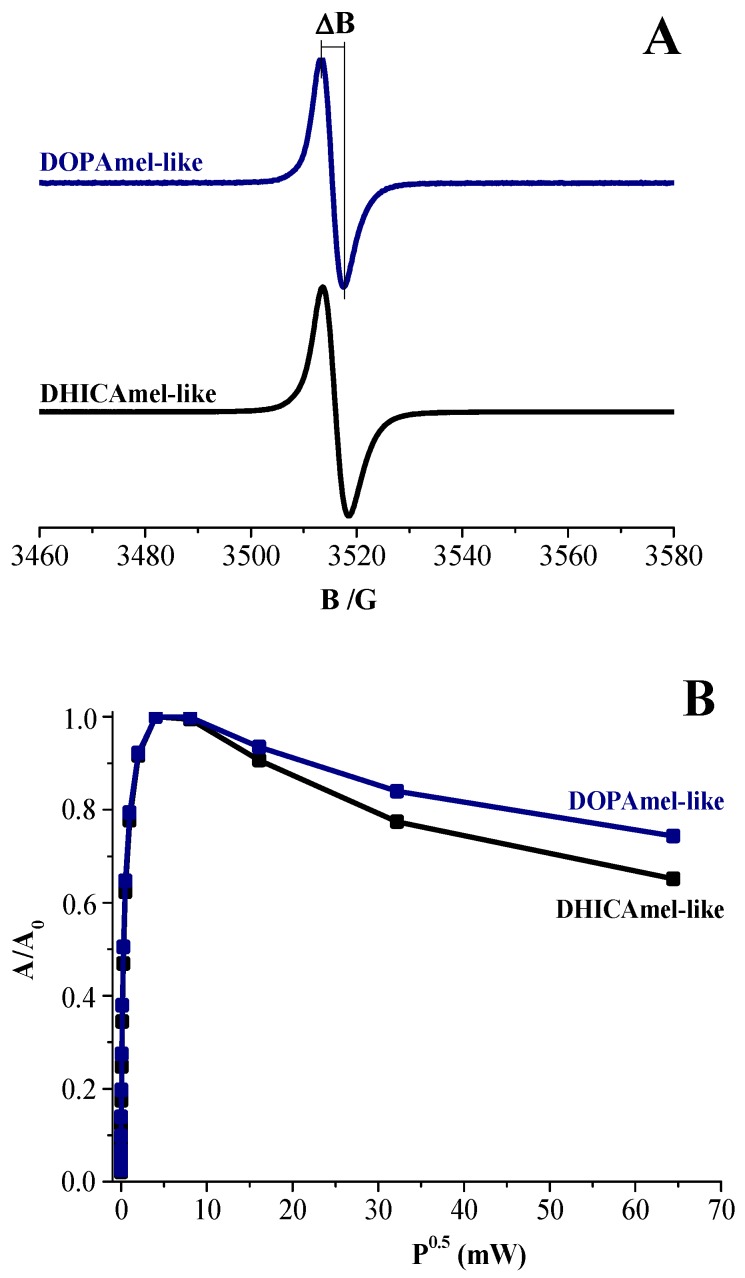
EPR spectra (**A**) and normalized power saturation curves (**B**) of hybrid DOPAmel-like (**A**) and DHICAmel-like (**B**) nanostructures precursors.

The chemical nature of obtained nanostructures was confirmed by EPR measurements performed following an experimental procedure recently used in the characterization of melanins [25,28,29], also providing significant information on the supramolecular properties of hybrid nanomaterials. EPR signals, as shown in Figure 4A, presented a very similar line shape: a single, roughly symmetric signal was detected at a g-factor value of 2.0030 ± 0.0003 for both DOPAmel-like and DHICAmel-like nanohybrids. A deeper analysis of EPR spectra showed that the DOPAmel-like spectrum was narrower than the DHICAmel-like one. The difference in line shapes was also corroborated by the quantitative determination of the signal amplitude (∆B), as reported in Table 1. This parameter, detectable by the experimental spectra as described in Figure 4A, was associated with the mean distance between the radical centers and furnished indirect information about the supramolecular organization. Particularly, the ∆B value determined from the DOPAmel-like spectrum resulted in being lower than that obtained from the DHICAmel-like spectrum, suggesting that the radical centers had a further spatial distribution probably ascribable to a different morphological organization of macromolecules within the hybrid nanostructures.

EPR spectra of all samples were also recorded to increase the incident microwave power. The obtained power saturation curves are shown in Figure 4B, plotting the normalized peak amplitude (A/A_0_) values as a function of the square root of the microwave power (P). A decrease in amplitude for higher microwave powers was obtained for both samples, which is typical of a homogeneous chemical and spatial distribution of radical centers. This result indicated that free radicals had similarly long relaxation times and were homogeneously located on the macromolecular backbone, similar to melanins [9,27,28].

However, EPR spectra were poorly influenced by chemical environmental conditions (Figure S5, Table S1), such as the pH, hydration and presence of multivalent metal ions (Zn^2+^) in saturating conditions, contrarily to what reported in previous papers [32,33,34,35,36], indicating that the comproportionation equilibrium of the dihydroxyindole moieties, which is a key physicochemical feature of any melanin, was suppressed. As widely assessed in previous studies [11,12,14,20], Ti–DHICA/DOPA as well Ti–citric acid complex was stably englobed within the final samples and was expected to inhibit the comproportionation equilibrium. Therefore, overall experimental evidences seem to suggest that the final obtained melanin-like nanomaterials significantly differ from melanin.

**Figure 5:**
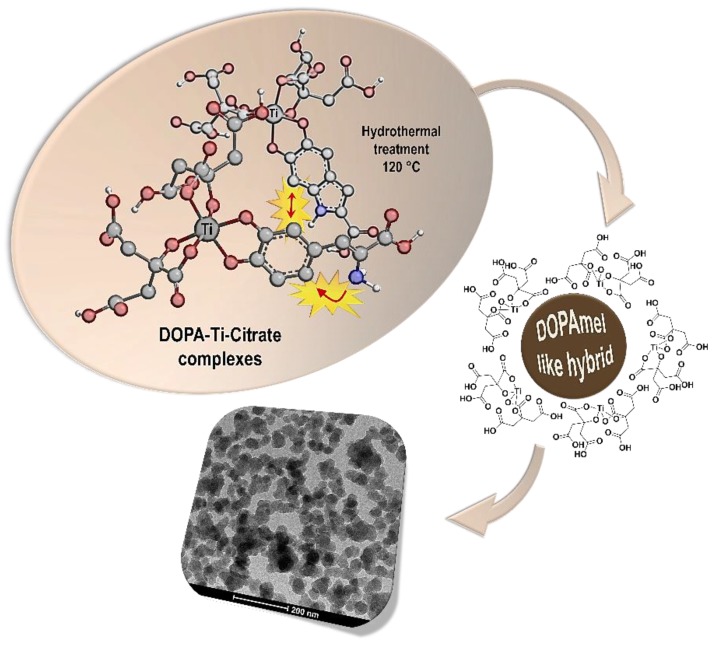
Schematic illustration of DOPAmel-like nanostructures’ formation mechanism.

Based on the physico-chemical experimental results, a possible formation mechanism can be proposed for DOPAmel-like nanostructures, as drawn in Figure 5.

Particularly, at low pH values, Ti coordination was completely saturated by citrate ions [37]. However, the stability of the Ti–citrate complex decreased with the pH increase, whereas the catechol moiety of DOPA showed a higher binding ability towards metal ions. Therefore, as soon as pH was raised up to 5.0, DOPA partially replaced citrate in the Ti coordination, forming a mono-catecholate/Ti complex as evidenced by the UV-Vis spectra reported in Figure 1. Moreover, the hydroxyl groups (-OH) in the citric acid moiety formed a supramolecular assembly among the ligated organic acid moieties via hydrogen bonding and hydrophobic interactions [38,39], resulting in the formation of a globular nanostructures.

Notably, unlike acetic acid, the citric acid chelating attitude interfered with the formation of Ti complexes along with the amino and carboxyl groups of DOPA which were consequently available to the oxidation process following the usual melanogenic pathway. Indeed, during the hydrothermal treatment, DOPA coupling occurred and catechol-metal complexes progressively evolved into covalent catechol-catechol intermolecular bonds templated by a citrate supramolecular cage, finally leading to pseudo-spherical nanosized structures.

### 3.2. Antimicrobial Tests

**Figure 6:**
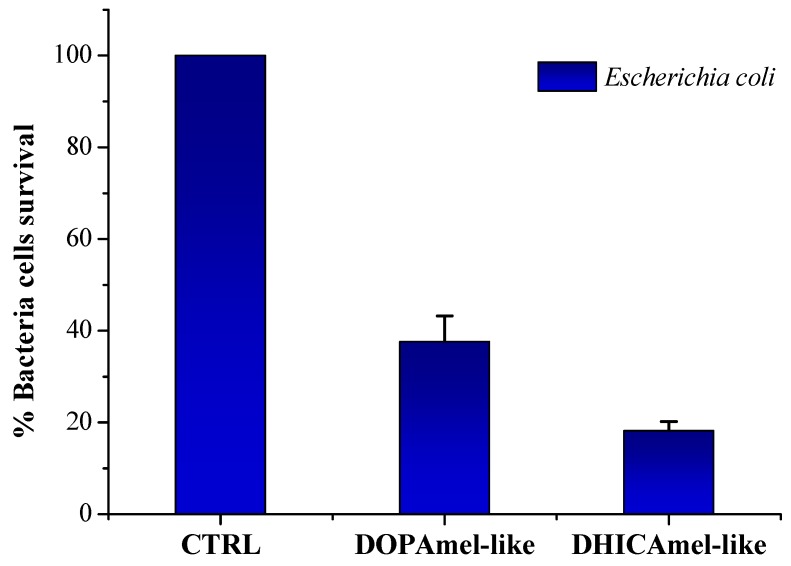
Antimicrobial activity of different nanostructures tested on the first day of synthesis, evaluated by colony count assay, against *Escherichia coli* DH5a strain. The percentage of bacterial survival is represented on the *y* axis. *E. coli* cells with any treatment represent the negative control (CTRL). The assays were performed for three independent experiments. *P* value was <0.05.

Finally, both DOPAmel-like and DHICAmel-like nanostructures have been tested for their antimicrobial activity, as previously described for hybrid melanin/TiO_2_ nanostructures [9]. A strong antimicrobial activity directed against the bacterial strain *Escherichia coli* is shown in Figure 6. Particularly, at a fixed concentration of 200 µg/mL, freshly prepared DOPAmel-like nanostructures showed a bacterial cell survival of 40%, with respect to a percentage of 20% detected for DHICAmel-like sample ones, showing a greater antibacterial activity. These results indicated a relative antibacterial improvement of DOPA-based nanosystems obtained by citric acid-modified synthesis with respect to those prepared by the acetic acid-based approach. On the other hand, an opposite behavior was observed for DHICA–based nanosystems obtained by citric acid-modified synthesis, showing a reduced biocide activity with respect to previously investigated DHICAmel/TiO_2_ nanostructures [9,11,12].

## 4. Conclusions

The overall spectroscopic and morphological results unveil the synergy between citrate–Ti(IV) and DOPA–Ti(IV) complexes in controlling DOPA polymerization, mediating and tuning its conversion to melanin-like oligomers. Notably, supramolecular assembly of Ti–ligated citrate moieties via hydrogen bonding and hydrophobic interactions provided a cage boundary where DOPA cyclization occurred and catechol–metal coordinative bonds were progressively replaced by irreversible catechol–catechol intermolecular bonds formed by the oxidation process. These reactions were selectively promoted during hydrothermal treatment. This thermal soft-polymerization process allowed morphological and size control during oxidative polymerization resulting in the formation of nanosized globular nanostructures, with marked antimicrobial efficacy. These findings will provide strategic guidelines to set up biomimetic processes, exploiting the catechol–metal complex to obtain hybrid melanin-like nanosystems with optimized multifunctional behavior for a wide range of technological applications.

## Figures and Tables

**Table 1:** Spectral parameters obtained by analysis of EPR spectra for hybrid DOPAmel-like and DHICAmel-like nanostructures. Estimated experimental uncertainties are ± 0.0003 on *g*-factor, ± 10% on spin-density, ± 0.2 G on ∆B.

Samples	*g*-Factor	ΔB	Spin-Density (×10^19^ spin/g)
**DOPAmel-like**	2.0030	4.2	0.065
**DHICAmel-like**	2.0032	4.9	1.2

## Supplementary Materials

The following are available online at https://www.mdpi.com/2313-7673/4/2/40/s1. Figure S1. XRD profiles of DOPAmel-like and DHICAmel-like nanostructures. Figure S2. TGA diagrams of DOPAmel-like and DHICAmel-like nanostructures. Figure S3. FTIR spectra of DOPAmel-like and DHICAmel-like nanostructures. Figure S4. UV-Vis spectra obtained after dissolution of DOPAmel-like and DHICAmel-like nanostructures in alkaline media. Figure S5. EPR spectra of DOPAmel-like and DHICAmel-like nanostructures at different conditions. Table S1. Spectral parameters of EPR spectra of DOPAmel-like and DHICAmel-like nanostructures at different conditions.
