# Supplementary material for: Citric Acid Tunes the Formation of Antimicrobial Melanin-Like Nanostructures
Source: Biomimetics (Basel). 2019 May 30;4(2):40. doi: 10.3390/biomimetics4020040 (PMC6630385; doi:10.3390/biomimetics4020040)
Supplement: Supplementary file 1 [file biomimetics-04-00040-s001.pdf]

Supporting Information

Citric acid tunes the formation of antimicrobial melanin-like nanostructures

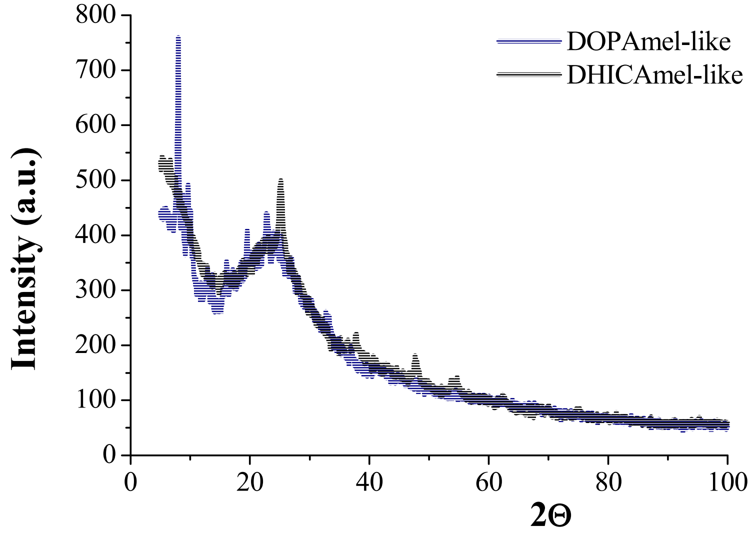

Figure S1. XRD profiles of DOPAmel-like and DHICAmel-like nanostructures.

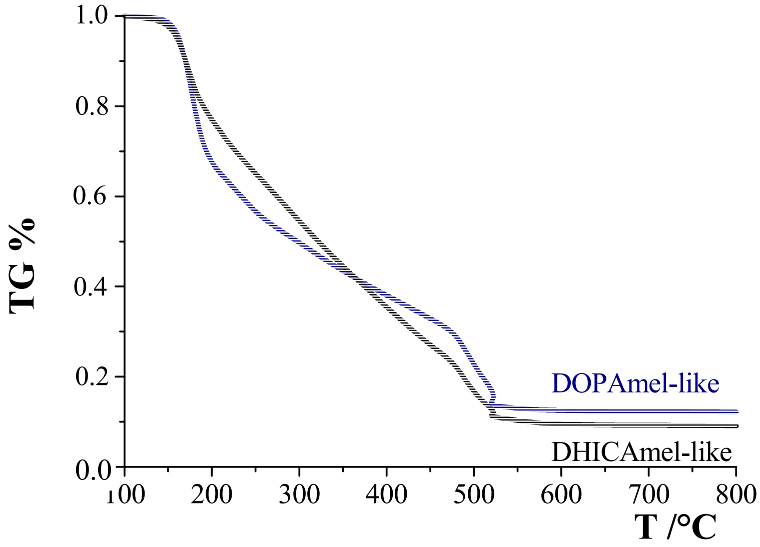

Figure S2. TGA diagrams of DOPAmel-like and DHICAmel-like nanostructures.

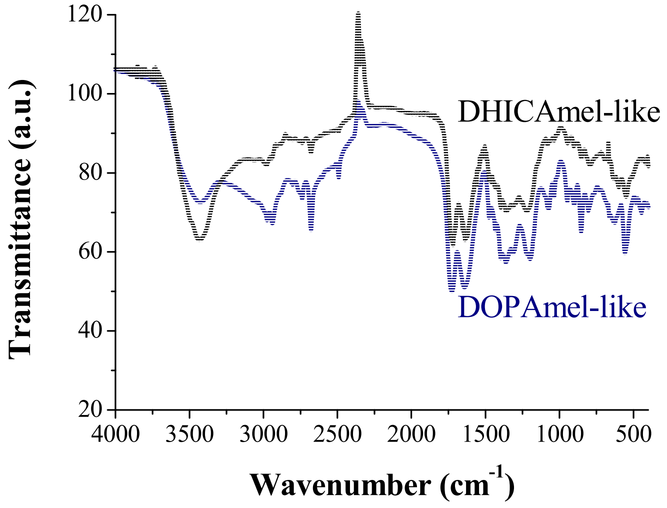

Figure S3. FTIR spectra of DOPAmel-like and DHICAmel-like nanostructures.

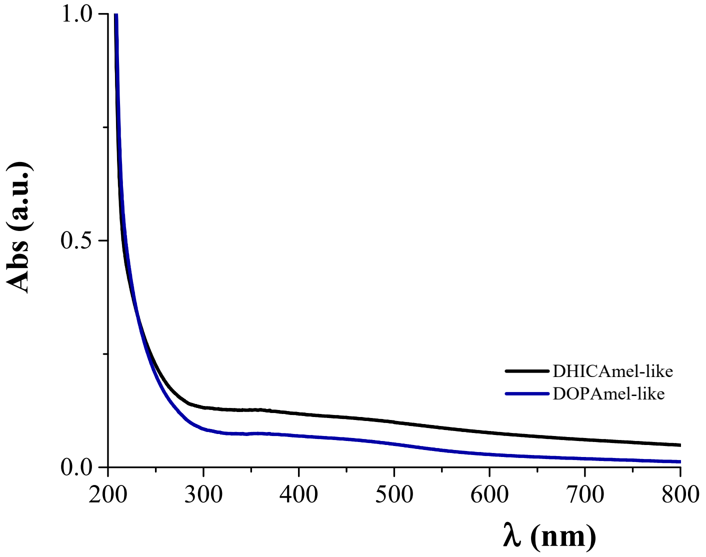

Figure S4. UV-vis spectra obtained after dissolution of DOPAmel-like and DHICAmel-like nanostructures in alkaline media.

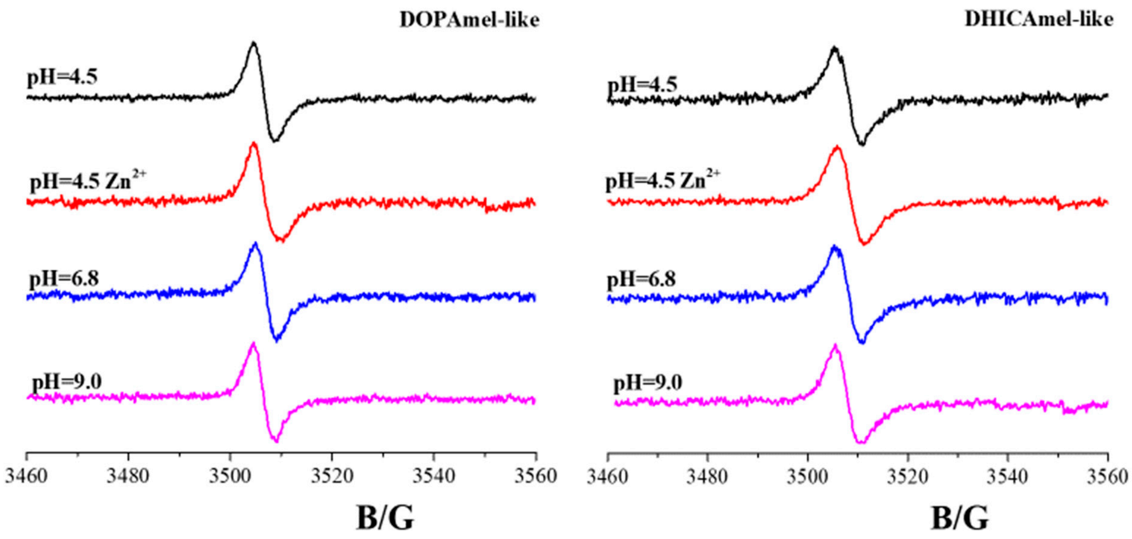

Figure S5. EPR spectra of DOPAmel-like and DHICAmel-like nanostructures at different conditions.

Samples	*g*-factor (±0.0003 G)	ΔB (±0.2 G)	Spin-density $\times 10^{19}$ spin/g (Err. ±10%)
**DOPAmel-like**	2.0030	4.2	0.065
**DOPAmel-like pH=4.5**	2.0030	4.4	0.013
**DOPAmel-like pH=4.5 (Zn^{2+})**	2.0035	5.2	0.059
**DOPAmel-like pH=6.8**	2.0027	4.1	0.024
**DOPAmel-like pH=9.0**	2.0027	4.2	0.037
**DHICAmel-like**	2.0032	4.9	1.200
**DHICAmel-like pH=4.5**	2.0027	5.2	1.010
**DHICAmel-like pH=4.5 (Zn^{2+})**	2.0029	5.7	1.350
**DHICAmel-like pH=6.8**	2.0028	5.3	1.105
**DHICAmel-like pH=9.0**	2.0027	5.2	1.215

Table S1. Spectral parameters of EPR spectra of DOPAmel-like and DHICAmel-like nanostructures at different conditions.
